# When voices can be cloned: vocal identity threat and occupational self-efficacy among singers in the era of generative AI audio

**DOI:** 10.3389/fpsyg.2026.1954386

**Published:** 2026-10-16

**Authors:** Jingjing Niu, Yinghao Qi, Xiaobin Li

**Affiliations:** 1 Department of Music Education and Musicology, Institute of Music, Theatre and Choreography, Herzen State Pedagogical University of Russia, Saint Petersburg, Russia; 2 St. Petersburg State Institute of Culture, Saint Petersburg, Russia; 3 Beihua University, Jilin, China

**Keywords:** audio deepfakes, creative labor, generative AI audio, occupational self-efficacy, singers, vocal identity threat, voice cloning

## Abstract

Generative AI voice cloning has advanced rapidly, yet its psychological and professional implications for singers remain underexplored. This review synthesizes empirical evidence concerning two central constructs: vocal identity threat, defined as the perceived erosion of the connection between voice and self, and occupational self-efficacy, referring to confidence in maintaining professional competence and value under technological uncertainty. Neurocognitive and behavioral evidence indicates that the human voice functions as a highly self-relevant identity cue supported by self-prioritized processing, body ownership, and multisensory integration. As AI-generated voice clones become increasingly humanlike, questions have emerged about how such technologies may affect singers' perceptions of vocal identity and professional value. Importantly, perceptual discrimination between human and AI-generated voices remains dependent on stimulus characteristics, listener factors, and the availability of prosodic and expressive cues. We argue that three distinctions are critical for interpreting the current evidence: timbral similarity should not be equated with artistic equivalence, increasingly humanlike output does not establish perceptual interchangeability, and the implications of AI may differ depending on whether it substitutes for identity-bearing vocal labor or scaffolds human-controlled performance and creation. Current evidence therefore provides an empirical basis for investigating potential AI-related threats to singers' vocal identity and occupational confidence, but direct singer-specific evidence remains unavailable. Future research should prioritize singer-specific quantitative studies employing longitudinal designs, ecologically valid voice-cloning scenarios, comparison conditions, and direct measures of identity threat and occupational self-efficacy.

## 1 Introduction

Generative AI audio has developed rapidly. Latent diffusion models, neural audio codecs, and neural vocoders drive this progress (Shen et al., 2024). These systems can generate natural speech and singing voices with high timbre similarity under few-shot or zero-shot conditions (Shen et al., 2024). This stage requires identification, anti-counterfeiting, and governance (Zhang et al., 2024). However, systematic empirical integration is lacking regarding how generative AI audio psychologically impacts human singers. Therefore, this article focuses on two core psychological constructs. The first is vocal identity threat. This refers to the perceived erosion of the bond between a person's voice and self-concept when facing an AI voice that can be copied, appropriated, or replaced. The second is occupational self-efficacy. This is a singer's subjective judgment of their ability to maintain professional competitiveness, complete occupational tasks, and cope with future work uncertainty (Schyns and von Collani, 2002). This issue is not purely theoretical, although direct evidence from singers remains unavailable. Ouyang (2025) provides related evidence from an adjacent domain of vocal performance rather than from singing itself. In a single-time-point mixed-methods study conducted at three Chinese universities, 414 undergraduate students enrolled in a Dubbing and Artistic Performance course completed measures of AI anxiety and performance appraisal in the context of creative dubbing tasks. Higher AI anxiety was associated with greater underestimation of expressive performance relative to external ratings and with lower external ratings. However, these external evaluations were provided by peers without professional training, themes concerning artistic identity were derived from interviews with only five students, and career concern was included as an item within the AI anxiety measure. The findings should therefore be interpreted cautiously as adjacent evidence concerning AI-related anxiety and vocal performance rather than as direct evidence from singers. Furthermore, research on self-voice processing points out a stable self-prioritization tendency for a person's own voice. The voice is not an ordinary tool. It is a highly self-relevant identity cue (Kirk and Cunningham, 2025). To our knowledge, no empirical study has yet directly examined singers' psychological responses to AI voice cloning. We therefore integrate available evidence from voice perception, vocal-performance education, music research, and adjacent creative domains to clarify three distinctions: timbral similarity vs. artistic equivalence, humanlikeness vs. perceptual interchangeability, and AI as substitution vs. AI as a scaffold.

## 2 Conceptual clarification and technical baseline

Generative AI audio refers to computational systems that generate acoustic representations or waveforms. These systems fit conditional distributions on large-scale speech and singing data based on statistical learning. They minimize prediction errors in neural networks. This is not a human learning process. Accordingly, TTS refers to text-to-speech synthesis. Voice conversion refers to changing the speaker's timbre while keeping the linguistic content. Voice cloning emphasizes copying the vocal identity of a specific individual. In singing scenarios, these results generally fall under the category of audio deepfakes (McGettigan et al., 2025). Importantly, singing voice identity involves more than timbral similarity alone. Singing-specific features such as vocal timbre, pitch control, vibrato, register, articulation, phrasing, dynamics, and expressive timing contribute to how a singing voice is produced and perceived (Sundberg, 1994; Gunjawate et al., 2018). These features are particularly relevant to voice cloning because reproducing a singer's recognizable timbre does not necessarily entail reproducing the broader expressive characteristics of their performance. NaturalSpeech 2 exemplifies this. This system combines a neural audio codec with a diffusion model. It achieves zero-shot speech and singing generation trained on 44,000 h of data. Current models can reconstruct voices that sound like a specific person with high fidelity under controlled conditions (Shen et al., 2024). Regarding the technical baseline of the threat, the equal error rate of the best system in the controlled track of SVDD 2024 dropped to 1.65 percent. High-quality singing deepfakes require specialized evaluation and countermeasures (Zhang et al., 2024). Human detection of AI-cloned voices is above chance but remains sensitive to stimulus and task characteristics. A meta-analysis by Diel et al. (2024) reported an average detection accuracy of 62.08% for audio deepfakes. Barrington et al. (2025) further found that listeners classified AI-cloned and authentic voices with approximately 60.8% accuracy, significantly above chance. Importantly, detection accuracy increased for longer and unscripted clips, and listeners most frequently reported relying on vocal inflection. These findings suggest that access to richer expressive information can facilitate discrimination between human and AI-generated voices. This point may be particularly relevant to singing, which contains richer melodic, rhythmic, prosodic, and expressive information. Chen et al. (2026), for example, showed that prosodic cues can strengthen perceptual boundaries between human speakers and AI voice clones, suggesting that discrimination depends partly on the acoustic and expressive information available to listeners. Earlier work on artificial singing voices similarly demonstrated that listeners can remain sensitive to differences in humanness and naturalness even when lyrics, melody, and rhythm are controlled (Kuriki et al., 2016). However, Kuriki et al. (2016) used Vocaloid-generated singing voices rather than cloned versions of the same singers' voices, with a small sample and stimuli generated using a single synthesis program. The study therefore demonstrates sensitivity to perceived humanness in artificial singing rather than the detectability of contemporary singing voice clones. These findings are particularly relevant to singers because vocal identity in singing involves not only timbral similarity but also expressive timing, pitch control, phrasing, vibrato, articulation, and other performance-related cues. Current evidence therefore suggests that contemporary voice clones are increasingly humanlike, while their perceptual equivalence to human singing voices remains unestablished.

## 3 Empirical evidence of vocal identity threat

Neurocognitive research indicates that one's own voice is processed as a highly self-relevant auditory cue rather than as an ordinary acoustic stimulus. Neuroimaging and EEG studies show reliable differentiation between self and other voices, supporting specialized self-referential voice processing (Kaplan et al., 2008; Hosaka et al., 2021; Iannotti et al., 2022). Research on the “rubber voice” illusion further suggests that perceived voice ownership can be altered when external vocal signals are closely synchronized with one's own behavior (Banakou and Slater, 2014; Zheng et al., 2011). Together, these findings support the identity relevance of self-voice processing, although they do not by themselves demonstrate that AI voice cloning produces identity threat.

### 3.1 Voice identity beyond singing

The relationship between voice and identity is not limited to performers. Research in augmentative and alternative communication (AAC), for example, shows that a synthetic voice may become part of how a person is recognized by others and how they present themselves socially. Weinberg et al. (2026) found that AAC users evaluated AI-generated voices not only in terms of acoustic fit, but also in relation to cultural and linguistic representation, belonging, self-recognition, and agency. This work broadens the present discussion by showing that voice identity can remain psychologically meaningful even when the audible voice is technologically mediated rather than biologically produced. The case of singing is nevertheless distinctive. For singers, the voice is simultaneously a means of self-expression, a trained bodily instrument, and a source of professional recognition and economic value. Thus, evidence from accessibility research helps establish the broader identity relevance of synthetic voice, while the occupational implications of voice cloning remain particularly salient in singing.

Building on broader identity-threat theory, which defines identity threat in terms of perceived potential harm to the value, meaning, or enactment of an important identity (Petriglieri, 2011), we use the term vocal identity threat to describe singers' perception that the valued connection between their voice and self may be undermined when that voice can be copied, manipulated, appropriated, or replaced by an external system. We therefore treat vocal identity threat as a singer-specific conceptual application of identity-threat theory rather than as an already established standalone psychological construct. Current evidence directly related to AI cloning mainly comes from two types of research. First, perception and social evaluation studies show that familiarity with the target voice affects responses to voice clones. Familiar listeners were able to distinguish cloned from recorded voices, whereas unfamiliar listeners were unable to distinguish them at the group level and sometimes rated cloned voices as favorably as or more favorably than the originals (Rosi et al., 2025). The familiarity effect further suggests that the implications of voice cloning may differ according to how familiar audiences are with a singer's voice: familiar listeners may be more sensitive to deviations from the original voice, whereas unfamiliar listeners may be less able to identify such differences. Importantly, participants showed no detection advantage for clones of their own voice compared with clones of a friend's voice, indicating that self-prioritized voice processing does not necessarily translate into superior conscious detection of one's own voice clone. Second, speech-based perception studies indicate that contemporary voice clones can reproduce convincing identity cues, although the degree of perceptual similarity varies across tasks, listeners, and stimulus characteristics (Barrington et al., 2025; Chen et al., 2026). These findings should be treated as supporting rather than direct evidence for singers because singing provides additional melodic, prosodic, and expressive cues that may facilitate human–AI discrimination (Kuriki et al., 2016). Nevertheless, the increasing capacity to reproduce recognizable vocal characteristics may be psychologically salient for singers and vocal students whose professional identity is closely associated with their voice. Their professional capital relies heavily on voice recognizability. Adjacent evidence from vocal-performance education is consistent with the possibility of such concerns: Ouyang (2025) found that students with higher AI anxiety tended to underestimate their expressive performance relative to external ratings and also received lower external ratings.

Existing evidence remains insufficient to determine whether audio deepfakes produce stronger identity threats than other forms of synthetic media. For the present review, the more relevant issue is that voice carries unusually strong self-referential and professional meaning for singers. Direct singer-specific evidence on psychological responses to AI voice cloning is currently lacking. Relevant evidence instead comes primarily from adjacent domains, cross-sectional studies, and one-time evaluations. This is insufficient to determine whether these initial concerns or reactions will persist or gradually habituate. Broader deepfake psychological impact research also remains methodologically heterogeneous and lacks substantial longitudinal evidence (Ching et al., 2025). Current evidence supports the identity relevance of self-voice processing and provides preliminary evidence that voice cloning may be psychologically salient. Whether this develops into persistent vocal identity threat, however, remains unclear, particularly with respect to duration, dose–response patterns, and long-term effects among professional singers.

## 4 Occupational self-efficacy among singers

Although singers remain the primary population of interest in this review, direct singer-specific evidence is currently unavailable. Evidence from adjacent vocal-performance contexts, other musicians, and creative professionals is therefore used cautiously to inform possible psychological and occupational implications for singers. This section focuses on whether singers and music creators believe they can rely on professional skills to sustain creation, performance, and livelihood tasks. This is occupational self-efficacy. Available negative evidence primarily concerns perceived professional threat and self-evaluation rather than direct measures of occupational self-efficacy. It does not manifest as a direct report that occupational self-efficacy has decreased by a specific percentage. For example, in an adjacent vocal-performance context, Ouyang (2025) found that higher AI anxiety was associated with greater underestimation of expressive performance relative to external ratings. Notably, Ouyang (2025) also reported that more frequent AI use was associated with lower AI anxiety and higher self- and externally rated performance, providing adjacent evidence consistent with the scaffolding side of the proposed framework. A survey by PRS for Music based on over 2,630 members showed that 79 percent of respondents worry about AI-generated music competing with human works. Also, 76 percent believe AI could harm creators' livelihoods. The 2025 UK Music creator survey showed that 66 percent of respondents agree that AI poses a threat to their career. These findings should not be interpreted as direct evidence of reduced occupational self-efficacy. Rather, they document broader perceptions of career threat and occupational insecurity that may create conditions under which self-efficacy becomes vulnerable.

Across the adjacent evidence reviewed here, the psychological implications of AI appear to vary depending on whether the technology substitutes for or supports human performance. Some studies report higher task-specific self-efficacy when AI is used as a collaborative scaffold rather than as a substitute output. A quasi-experimental study by Ou et al. (2025) on 40 violin performance students found that the experimental group using AI-assisted practice applications scored significantly higher on the performance self-efficacy post-test. The effect size reached d = 1.18. Wang and Zhang (2025) found that AI use was positively associated with musical creativity, with musical self-efficacy statistically mediating this association. Adjacent evidence from non-vocal creative tasks is consistent with this interpretation. McGuire et al. (2024) found in a poetry writing experiment that participants co-creating with AI had significantly higher creative self-efficacy than those editing AI drafts. Their expert creativity scores were no longer significantly lower than the pure human creation condition. Observed differences in self-efficacy appear to vary according to the degree of human control, choice, and process ownership (Ou et al., 2025; Wang and Zhang, 2025; McGuire et al., 2024).

The apparent inconsistency across studies becomes more coherent when differences in creator role, AI use, and outcome measurement are considered. Record producers and recording engineers, for example, often use AI for audio restoration, demo production, and mastering (UK Music, 2025), whereas songwriters, composers, and performers whose professional value depends more directly on original attribution may perceive greater replacement pressure. For singers whose professional identity is closely tied to a recognizable personal voice, this distinction may be particularly important. Responses also vary with the role assigned to AI: editing ready-made AI outputs may provide less perceived agency than interactive co-creation. A further source of variation lies in study design. Industry surveys generally assess broad occupational threat, whereas experimental studies tend to measure short-term, task-specific self-efficacy. These measures describe related but different aspects of creators' responses to AI. Broader occupational research further suggests that responses to AI vary across cultural and demographic contexts. In a mixed-method experimental study of 480 participants from the United Kingdom and Mexico, Xavier et al. (2025) found differences in receptivity to AI integration across cultural groups, while effects on creative self-efficacy were not uniformly positive. These findings further caution against assuming a single direction of AI effects across populations.

Current evidence remains insufficient to establish that AI-related career concerns translate into persistent reductions in singers' occupational self-efficacy. Negative evidence mainly comes from cross-sectional self-reports or industry surveys that document perceived career threat rather than occupational self-efficacy itself. Positive evidence largely derives from small-sample quasi-experiments or laboratory tasks measuring musical or creative self-efficacy, which limits generalizability to long-term occupational beliefs. Current findings therefore suggest that AI-related career concerns may be associated with reduced professional security or confidence among some music creators, but these associations are unlikely to be universal and may depend on creators' roles, the function assigned to AI, and the outcome being measured (Ouyang, 2025; Ou et al., 2025; Xavier et al., 2025).

## 5 Discussion: pointing out gaps in empirical data

Three distinctions emerge from the evidence reviewed here. First, reproducing a singer's vocal timbre should not be equated with reproducing the broader artistry of singing, which also involves phrasing, dynamics, articulation, vibrato, and expressive timing. Second, increasingly humanlike synthetic voices should not be treated as perceptually interchangeable with human voices, because discrimination varies with stimulus characteristics, listener familiarity, and the expressive information available. Third, we propose a substitution–scaffolding framework for understanding the possible psychological implications of generative AI for singers. AI may have different implications when it substitutes for identity-bearing vocal performance than when it supports human-controlled practice, creation, or production. This framework is a conceptual synthesis based primarily on adjacent evidence from voice perception, vocal-performance education, music education, and creative work rather than on direct empirical studies of professional singers.

The evidence reviewed here does not support treating “fear” as a single, uniformly measured psychological outcome. Different studies operationalize related responses in different ways. Ouyang (2025), for example, examined AI anxiety and artistic self-evaluation, whereas the PRS for Music and UK Music surveys assessed perceived competition, livelihood concerns, and career threat. Studies of voice cloning, by contrast, have primarily examined perceptual discrimination, familiarity, and social evaluation rather than psychological threat itself. These outcomes operate at different levels and should not be collapsed into a single estimate of fear among singers. What the available literature does show is that several conditions relevant to identity and occupational concern are present: the human voice is strongly self-relevant, many music creators report concern about AI-related competition and livelihood, and cloned voices can reproduce recognizable identity cues even though human–AI discrimination varies across listeners and stimuli. For singers, these findings make vocal identity threat and occupational insecurity plausible subjects of empirical study, but they do not yet establish a stable psychological syndrome or a long-term population-level effect.

The limits of the current evidence are equally important.

Current evidence from adjacent vocal-performance and music contexts indicates AI-related anxiety, occupational concern, and variation in short-term ability evaluations. These studies do not establish whether such responses persist over months or years. This distinction matters because existing research also shows that AI does not always weaken self-efficacy. Ou et al. (2025) found that AI-assisted practice significantly improves musical performance self-efficacy. The between-group effect size on the post-test reached d = 1.18. The available evidence does not indicate that AI inevitably harms singers. The psychological implications of AI may differ depending on whether it is experienced as a substitute or a scaffold. When AI reproduces identity-bearing properties of a singer's voice, it may be experienced as a threat. When AI supports practice while preserving human control, it may support perceived competence and self-efficacy. Claims about permanent existential fear are currently speculative. They are not conclusions proven by longitudinal empirical evidence. Broader deepfake literature supports this cautious attitude. The 22 empirical studies included by Ching et al. (2025) show that deepfake psychological impact research is generally early. It has high methodological heterogeneity and lacks long-term tracking. Looking beyond singing also helps clarify which parts of this problem may generalize and which may not. Concerns about authorship, agency, substitution, and professional value are shared with other creative domains. In poetry, for example, the effects of AI on creative self-efficacy vary with the role retained by the human creator rather than simply with the presence of AI (McGuire et al., 2024). Accessibility research presents a different relationship between synthetic voice and identity: for AAC users, a well-matched synthetic voice may support self-representation and agency rather than threaten them (Weinberg et al., 2026). Singing sits between these cases. Like other creative work, it raises questions of authorship and replacement, but the singer's voice is also embodied, individually recognizable, and directly tied to professional performance. Findings from other domains can therefore inform mechanisms such as agency and identity alignment, but they should not be assumed to transfer directly to singers.

Future research needs to move from broad assumptions about technological threat toward singer-specific questions that can clarify when and why voice cloning becomes psychologically consequential. One priority is to identify which features of singing are most closely tied to vocal identity. For example, future studies could examine whether cloning vocal timbre produces the same response as reproducing more expressive characteristics such as phrasing, vibrato, dynamics, articulation, and prosodic variation. It is also important to determine whether professional singers are more sensitive than non-singers to subtle differences between human and AI-generated singing, and whether such sensitivity varies across singing styles, levels of expertise, or familiarity with the target voice. A second priority is to distinguish between AI as a substitute for identity-bearing vocal performance and AI as a supportive tool for practice, composition, or production. Finally, longitudinal research is needed to determine whether initial concerns about vocal identity and professional value persist, diminish, or change as singers gain greater experience with generative AI. These directions would help clarify whether voice cloning produces only short-term technological unease or more enduring changes in singers' vocal identity and occupational self-efficacy.

## 6 Conclusion

Existing research suggests that the implications of generative AI audio for singers extend beyond copyright and labor-market concerns to questions of vocal identity and professional confidence. Existing data indicate that the voice has high self-relevance. High-quality voice cloning has become increasingly humanlike, although the extent to which listeners distinguish AI-generated from human voices depends on stimulus characteristics, listener factors, and expressive or prosodic information (Barrington et al., 2025; Chen et al., 2026). AI-related anxiety and discrepancies between self- and externally rated performance have been observed in an adjacent vocal-performance education context (Ouyang, 2025), while occupational threat perceptions have been reported in broader music-industry surveys (PRS for Music, 2026). Taken together, the available evidence provides an empirical basis for treating vocal identity and occupational confidence as plausible concerns for future singer-specific research, although direct empirical evidence from singers is currently lacking.

However, current evidence cannot support the stronger claim that singers will fall into irreversible existential fear long-term. The most urgent task for this field is not to produce more purely theoretical stance papers. It is to conduct more rigorous longitudinal quantitative research, ecological situational experiments, and specialized tracking studies targeting professional singers. Such research is needed to determine whether generative AI produces primarily short-term unease or contributes to more persistent changes in singers' vocal identity, professional confidence, and occupational self-efficacy.
